# Novel Melatonin-Blocking Complex Helps Control Body Rhythms

**DOI:** 10.1371/journal.pbio.1001348

**Published:** 2012-06-19

**Authors:** Janelle Weaver

**Affiliations:** Freelance Science Writer, Glenwood Springs, Colorado, United States of America

## Abstract

Dopamine and adrenergic receptor complexes form under a circadian-regulated cycle and directly modulate melatonin synthesis and release from the pineal gland.

**Figure:**
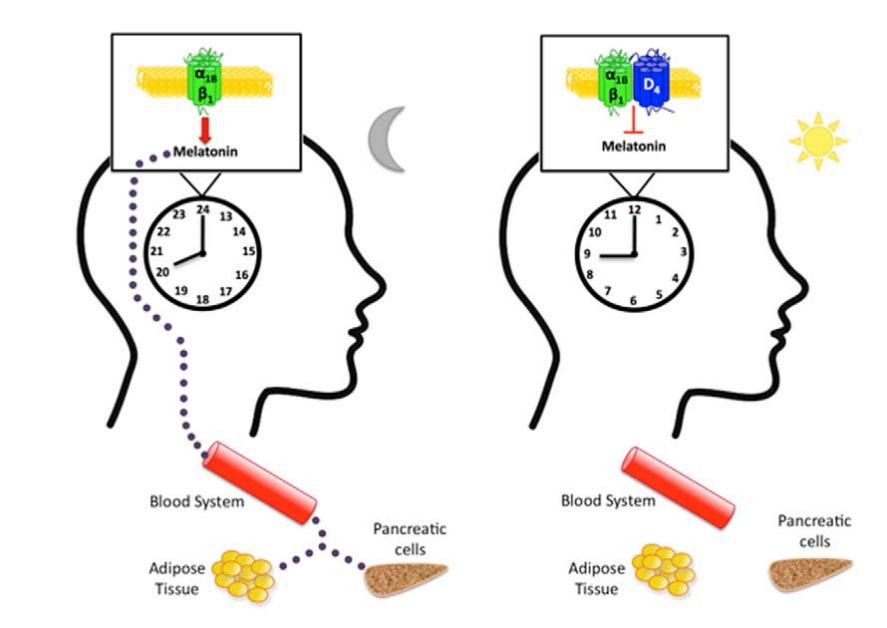
Melatonin production regulated by adrenergic receptors in the pineal gland can modulate circadian cycles of the body. The dopamine D4 receptor can inhibit melatonin release by forming circadian-controlled complexes with adrenergic receptors.

Circadian rhythms are daily cycles that control our sleep-wake patterns, hormone release, and body temperature, and are found in virtually all life forms. Because our internal clock runs just over 24 hours, our bodies set it to match day length using environmental cues such as light. Information about light levels travels from the eyes to a brain structure called the pineal gland, which adjusts the internal clock by secreting melatonin at night. The synthesis and release of this sleep-inducing hormone is known to be regulated by a class of cell-surface proteins called adrenergic receptors. However, it has been unclear exactly what limits the daytime rates of melatonin production.

This week in *PLoS Biology*, a team led by Peter McCormick of the University of Barcelona reports that adrenergic receptors form complexes with D4 dopamine receptors to block melatonin production during the day. The levels of these complexes (called heteromers) rise and fall in a circadian pattern—a phenomenon that has never before been reported for receptor heteromers. These findings elucidate the previously unknown role of D4 receptors in the pineal gland, and provide evidence for a novel molecular mechanism for controlling melatonin production in mammals.

In the study, McCormick and his collaborators detected the D4-adrenergic receptor complexes in cells isolated from the rat pineal gland. After attaching DNA strands to distinct antibodies that bind to D4 receptors and adrenergic receptors, they found that the two different receptors were close enough for the DNA strands on their respective antibodies to interact. This interaction occurred in pineal glands extracted just after sunrise but not at sunset, suggesting that the complexes form at the start of the light period, but their numbers diminish by the end of the day.

To examine the functional role of the D4-adrenergic receptor complexes, McCormick and his team treated pineal glands with compounds that activate both types of receptors. Stimulation of D4 receptors interfered with the ability of adrenergic receptors to activate their target signaling pathways (ERK 1/2 and Akt/PKB) and thereby increase melatonin synthesis and release. This inhibitory effect occurred in pineal glands extracted at sunrise, when the complexes were present in the tissue, but not in pineal glands removed at sunset, when only adrenergic receptors were expressed.

The newly identified complexes fit the classic definition of a heteromer because they take on biochemical and functional properties that are different from those of their individual units. Because these heteromers keep melatonin at low levels until nighttime, they could explain how the pineal gland is able to maintain precise control over the body's circadian rhythms.

**González S, Moreno-Delgado D, Moreno E, Pérez-Capote K, Franco R, et al. (2012) Circadian-Related Heteromerization of Adrenergic and Dopamine D_4_ Receptors Modulates Melatonin Synthesis and Release in the Pineal Gland. doi:10.1371/journal.pbio.1001347**

